# Cable energy function of cortical axons

**DOI:** 10.1038/srep29686

**Published:** 2016-07-21

**Authors:** Huiwen Ju, Michael L. Hines, Yuguo Yu

**Affiliations:** 1 School of Life Science and the Collaborative Innovation Center for Brain Science, Center for Computational Systems Biology, Fudan University, Shanghai 200433, China; 2 Department of Neuroscience, Yale University School of Medicine, New Haven, CT 06520, USA

## Abstract

Accurate estimation of action potential (AP)-related metabolic cost is essential for understanding energetic constraints on brain connections and signaling processes. Most previous energy estimates of the AP were obtained using the Na^+^-counting method, which seriously limits accurate assessment of metabolic cost of ionic currents that underlie AP conduction along the axon. Here, we first derive a full cable energy function for cortical axons based on classic Hodgkin-Huxley (HH) neuronal equations and then apply the cable energy function to precisely estimate the energy consumption of AP conduction along axons with different geometric shapes. Our analytical approach predicts an inhomogeneous distribution of metabolic cost along an axon with either uniformly or nonuniformly distributed ion channels. The results show that the Na^+^-counting method severely underestimates energy cost in the cable model by 20–70%. AP propagation along axons that differ in length may require over 15% more energy per unit of axon area than that required by a point model. However, actual energy cost can vary greatly depending on axonal branching complexity, ion channel density distributions, and AP conduction states. We also infer that the metabolic rate (i.e. energy consumption rate) of cortical axonal branches as a function of spatial volume exhibits a 3/4 power law relationship.

Estimation of the metabolic cost of action potential (AP) generation and propagation is vital for the construction of energy budgets for single neurons^1^ and for the whole brain^2^,^3^,^4^. Such estimates reveal computational rules such as optimal trade-offs between metabolic constraints and neural coding performance^5^,^6^,^7^,^8^,^9^,^10^,^11^ and improves the interpretation of functional magnetic resonance imaging data^12^,^13^,^14^. In most previous studies, the metabolic cost of APs was based on Na^+^ influx, which was then converted to the total ATP required by Na^+^/K^+^-ATPase pumps to restore ion gradients after an AP. Prior to direct recordings from mammalian neurons, the overlap of Na^+^ influx and K^+^ efflux during an AP was thought to be similar to that of the squid giant axon^15^. That is, the amount of Na^+^ was estimated to be fourfold the theoretically minimal amount of Na^+^ entry needed to produce the voltage change during an AP. However, recent experiments have revealed that during AP propagation, the ratio of the actual Na^+^ quantity to the theoretical minimum, or the excess Na^+^ entry ratio^15^,^16^, is much lower (e.g., 1.3 at mossy fiber boutons of hippocampal granule cells^17^) than the value of 4 that has been calculated for the squid axon^15^,^16^ and ranges from 1 to 2.4 for different subcellular compartments of the same cortical pyramidal neuron^1^. This ratio has been shown to be affected by temperature^18^ and ion-channel kinetics^1^,^17^. However, the Na^+^-counting method is controversial because it underestimates the metabolic costs for neurons in which ions other than Na^+^ and K^+^ also play key roles in AP generation. Moreover, the Na^+^-counting method cannot provide a complete explanation why the AP-propagation-related metabolic costs or energy efficiency varies among subcellular components within a neuron.

To address these shortcomings, energy estimation based on the electrochemical energy function was first performed using single-compartment Hodgkin-Huxley (HH) neuron models^19^,^20^,^21^. However, no study has analyzed the metabolic costs associated with AP propagation using more realistic, multi-compartment neuron models. Additionally, the effects of axonal geometry and ion channel distribution on energy consumption have not been systematically investigated. To address these issues, we derive the electrochemical energy function for the cable model of a HH-type cortical axon and then use the function to calculate energy consumption for unbranched axons and axons with several degrees of branching (branching level, BL). We found that the energy associated with AP conduction varies nonlinearly along an axon. Furthermore, the energy consumption rate of the entire branched axon scales as the 3/4 power of axonal volume, just as the metabolic rate of an entire organism scales with its body mass in many biological processes^22^,^23^,^24^. Thus, energy consumption may be profoundly affected by branching complexity, non-uniform ion channel distributions and AP conduction states.

## Results

### The Cable Energy Function for a Cortical Axon

**Figure 1:**
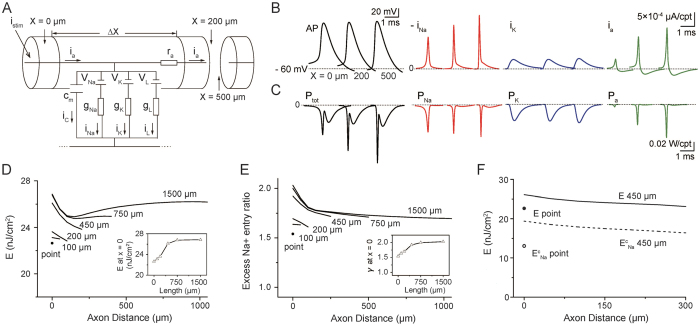
Effect of Axonal Length on AP-related Energy Consumption and Efficiency. (**A**) Cable model of a Hodgkin-Huxley-type cortical axon, where axial current, i_a_, flows through axial resistance, r_a_, within a uniform cylinder. The membrane currents consist of i_C_, i_K_, i_Na_, and i_L_, through c_m_, g_K_, g_Na_ and g_L_, respectively. V_K_, V_Na_ and V_L_, Nernst potentials. APs (60 Hz) were initiated by a stimulating current (19.1 μA/cm^2^, 1 s) at one end of the uniform axon (X = 0 μm) at 37 °C.(**B**) Traces of AP (black), -i_Na_ (red, inverted Na^+^ current), i_K_ (blue) and i_a_ (green) in the compartments of a uniform axon as in (A) (1.5 μm in diameter, 1000 μm in axonal length, 50 μm in compartmental length (Δx), unmyelinated) at X = 0, 200 and 500 μm. (**C**) Traces of the total energy consuming power (black, P_tot_ = P_Na_ + P_K_ + P_a_ + P_L_) and its main components, i.e., the energy consuming power of the sodium conductance (red, P_Na_ = i_Na_ × (V − V_Na_)), potassium conductance (blue, P_K_ = i_K_ × (V − V_K_)), and axial conductance (green, P_a_ = 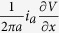), in the compartments at X = 0, 200, and 500 μm. P_L_ is the leak conductance (negligible). See also Figure S2. (**D**) Distribution of energy cost per unit membrane area per AP along axons of different lengths (same diameter, APs firing at 60 Hz, at 37 °C). Note that the longer the axon, the higher the energy cost at the same distance. Inset, increase of the energy cost at the AP initiation site with axonal length. (**E**) Distribution of the excess Na^+^ entry ratio (γ), i.e., the ratio of the total Na^+^ flux during an AP to the theoretically minimal Na^+^ load needed to generate the upstroke of an AP, along axons of different length. Note that the longer the axon, the higher the γ value at the same distance. Inset, increase of the value of γ at the AP initiation site with axonal length. (Theoretical minimum Na^+^ load: Na^+^ flux integrated from dv/dt = 20 to dv/dt = 0.) (**F**) Comparison of the energy calculation based on Na^+^ counting (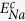) to the one based on the energy function (**E**). See also Figure S2.

To investigate the energy consumption associated with AP propagation, we return to the cable theory^25^ that underlies our HH-type cortical axon model^26^, which was constructed based on experimental measurements^27^,^28^,^29^,^30^. The model is conveniently represented as a multi-compartment equivalent circuit (Fig. 1A). Based on the cable equation that describes how ion currents flow along the cable (Methods Equation 4) as well as analysis of the electrochemical energy in the equivalent circuit, we derived the electrochemical energy function for the cable model (see Methods for detail),


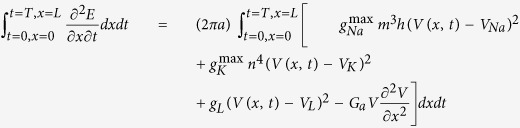


this equation describes the AP-related energy consumption rate per unit membrane area (cm^−2^s^−1^) at any axonal distance and any time. The individual terms on the right-hand side of the equation represent the contributions of the sodium, potassium, leak, and axial currents, respectively. Calculations based on this function distinguish between the contributions of each item toward total energy consumption.

### Effect of Axonal Length on AP-related Energy Consumption and Efficiency

Next, we applied the above energy function to estimate the distribution of energy cost along a single-cable axon (Fig. 1A). In the HH-type cortical axon model that is based on experimental data^18^,^30^,^31^, AP propagation (60 Hz, at 37 °C) was simulated by injecting a steady current (19.1 μA/cm^2^, 1 s) at one end of the axon (Fig. 1A; simulations in this paper were run in MATLAB). From recordings of APs, transmembrane ion currents, and axial currents at different axonal distances (Fig. 1B), we calculated the energy consumption associated with each ionic current (P_ion_ = i_ion_ × (V − V_ion_), Fig. 1C, Figure S2) as well as the total energy cost 
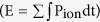
 as a function of axon length (see Fig. 1D). Although the Na^+^ current (i_Na_) amplitude during an AP increased with distance, the width of i_Na_ decreased (Fig. 1B, red), resulting in a reduction in total Na^+^ influx and the energy consumed by the sodium conductance (integration of the metabolic consuming power (P_Na_ = i_Na_ × (V − V_Na_)) of the sodium conductance over the duration of an AP, Fig. 1C, red). In contrast, the K^+^ current (i_K_) almost leveled off along the axon (Fig. 1B, blue), making the metabolic consuming power of the potassium conductance (P_K_ = i_K_ × (V − V_K_)) stable (Fig. 1C, blue). Additionally, the amplitude of the axial current (i_a_) rose sharply along the axon, resulting in a considerable increase in the amount of energy consumed by the axial conductance (integration of 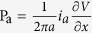).

To study the effect of axon length on energy consumption, we carried out simulations of unbranched cable axons ranging from 0 (point neuron model) to 1,500 μm in length (with the same diameter, AP firing at 60 Hz, at 37 °C). As shown in Fig. 1D, the energy cost along an axon of any length is generally highest at the AP initiation point, decreases sharply in the initial 200 μm, increases slowly and finally saturates at a value below that of the initial section. More crucially, the longer the axon, the higher the energy cost per unit membrane area per AP at the same distance (Fig. 1D). Even more importantly, in the cable model, the cost at the AP initiation site (E_int_) can be up to 15% higher than the cost in the point model (Fig. 1D, inset), indicating that more energy is needed to promote AP conduction across longer axons than across shorter ones. By analyzing the components that contribute to the energy consumption, we found that the higher cost in the longer axons was due to the higher cost of the axial and sodium conductances (see Fig. 1B,C), which is reasonable because AP propagation in longer axons demands a larger Na^+^ influx and a larger axial current.

We also examined how axonal length affects energy efficiency, as measured by the excess Na^+^ entry ratio (γ). A γ value greater than 1 indicates excess Na^+^ influx, and larger γ values, in turn, reflect a less efficient use of energy. As illustrated in Fig. 1E, the AP initiation location of the axon had the lowest efficiency, with values that ranged from 1.5 to 2; the longer the axon, the lower the efficiency at the same distance. In particular, the decrease in efficiency at the initiation site was up to 33% greater than the value of 1.5 obtained using the point model (note a value of 1.3 is obtained using the Na^+^ counting method), see Fig. 1E inset, indicating that long axons will pay the price of reduced efficiency.

To compare the energy-function approach with the traditional Na^+^-counting method, we applied both approaches to the cable model and the point model. Figure 1F shows that the cost estimated using Na^+^ counting was significantly lower in both a cable model with a length of 450 μm (27% lower) and in a point model of the same length (42% lower), suggesting that the Na^+^-counting method severely underestimates metabolic consumption. The discrepancy of the two results is calculated as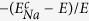, where 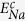 and *E* are the results of the sodium counting method and our approach, respectively. Our results show that factors including spiking rate, length of cable, temperature and gmax have limited effects on the discrepancy (<10%), indicating that the discrepancy is quite stable. See Discussion for detailed results.

### Effect of AP Conduction State on AP-related Energy Consumption

**Figure 2:**
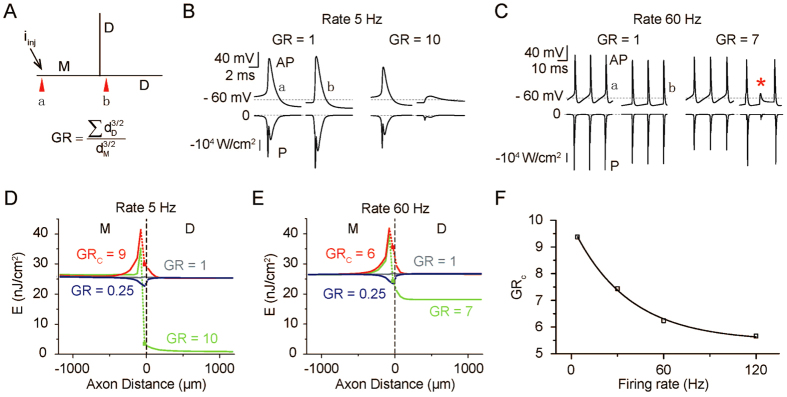
Effect of AP Conduction State on Energy Consumption. (**A**) Left, schematic diagram of a parent axon (**M**, the branch along which the propagating AP approaches the branch point) connected with a pair of identical child branches (**D**). d_M_ and d_D_ are the diameters of **M** and **D**, respectively. d_M_ remained 0.75 μm, and d_D_ was adjusted according to GR (geometrical ratio). **M** and **D** were both 250 μm in length. (**B**,**C**) Time courses of APs (top) and the energy consuming power (bottom) at the sites indicated by red arrowheads in (**A**) when AP conduction through the branch point was reliable (left) and unreliable (right), respectively. In (**B**), AP spike rate of 5 Hz; GR ≤ 9, reliable state; GR ≥ 10, unreliable state. In (**C**), AP spike rate of 60 Hz; GR ≤ 6, reliable state; GR ≥ 7, unreliable state; red asterisk indicates propagation failure. (**D,E**) Variation in energy cost per unit membrane area per AP along the axon (branch point is at X = 0) at four different GR values when the AP spike rate is 5 Hz (**D**) and 60 Hz (**E**). GR_c_ represents the critical GR, i.e., the largest GR for successful conduction. Note that in the child branch (X > 0), the energy cost per unit membrane area per AP was much lower in unreliable propagation states. (**F**) GR_c_ decreases exponentially with AP frequency.

Neurons exhibit various axonal branching patterns. Different AP conduction states (i.e., successful, blocked, and reflected conduction) have been observed at branch points in different types of neurons^32^. Factors that affect AP propagation through branch points include the geometry of the branches connected at the branch point^33^,^34^,^35^, the AP spike rate^32^,^36^,^37^ and the temperature^38^. To compare the energy costs of different AP conduction states, we examined a model consisting of a parent axon connected with a pair of identical branches (Fig. 2A). The parent axon is stimulated at the proximal end so that it generates APs at a controlled spike rate (5 or 60 Hz) at 37 °C. We determined the smallest child diameter at which AP propagation failure occurred for at least one AP in the train. Consider the geometrical ratio (GR)^39^,^40^, which is defined as


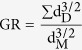


where *d*_*D*_ and *d*_*M*_ are the diameters of the child branches and the parent axon, respectively. Figure 2B illustrates the influence of the GR on APs (top) and energy consuming power (bottom) at the initiation site (a) and at 25 μm after the branch point (b) for a firing rate of 5 Hz. Successful propagation into child branches was observed when the GR was ≤9; however, when the GR reached 10, conduction failed at the branch point, indicating that the critical GR (GR_c_), or the largest GR for successful conduction, is between 9 and 10. Similar effects of GR on propagation were observed with an AP spike rate of 60 Hz but with a lower GR of 7 (Fig. 2C).

Figure 2D,E show the energy cost along the axon for two spike rates. For AP conduction through the branch point, less energy is used when the GR is small, while a larger GR generally requires more energy during the transition. For GR values greater than the critical value GR_c_, AP propagation failed in the child branch, and very little energy is consumed by the child branches. Actually, the rapid change in energy cost around the branch point at GR ≠ 1 (blue, red, and green) was mainly caused by AP reflection around the branch point. This phenomenon was not observed at GR = 1 because at that time, the APs were propagating in a branching system equivalent to a homogeneous parent axon in accordance with Rall’s 3/2 power law^40^. Our simulations predict that the GR_c_ decreases nonlinearly with increasing AP firing rate (Fig. 2F).

### Distribution of AP-related Energy Consumption in an Axonal Branching Tree

**Figure 3:**
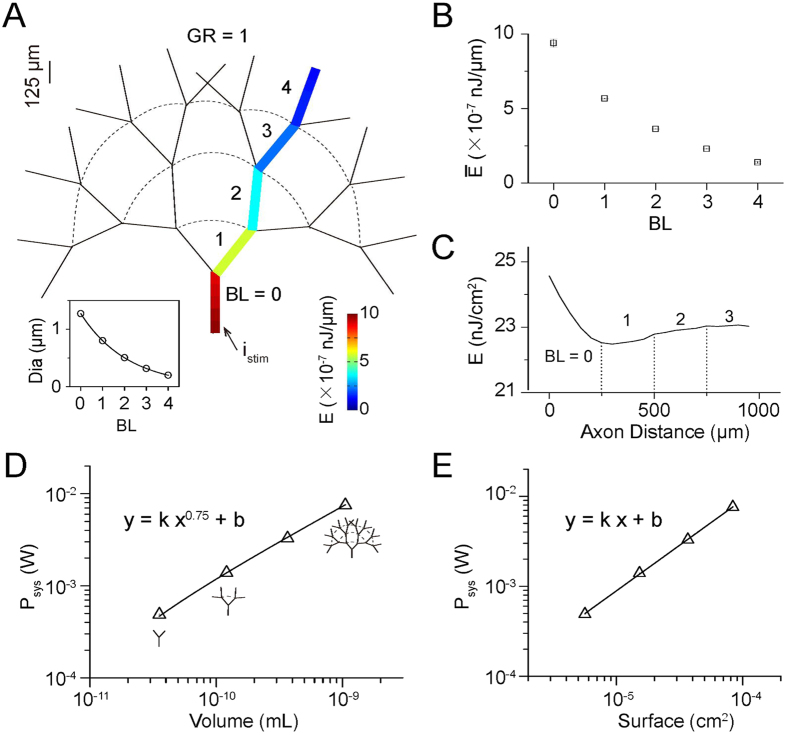
Distribution of AP-related Energy Consumption in Axonal Branching Trees. (**A**) Color-coded schematic diagram of an axonal branching tree with four branching levels (BLs). Colors indicate the energy cost per unit length per AP. APs (4 Hz, 37 °C) were elicited by the stimulating current (250 μA/cm^2^, 1 s) at the end of the branch at BL = 0. Inset, branch diameter (Dia) versus BL; GR = 1 for the diameter of branches of adjacent BLs; the diameter of the branch at BL = 0 and BL = 4 was 1.27 μm and 0.2 μm, respectively. Length of each branch, 250 μm. The connected axonal branches are not shown as perpendicular to each other (as are real axonal branches) for the sake of visual clarity. (**B**) Average energy cost per unit length per AP of each BL decreased with BL in the axonal branching tree shown in (**A**). Error bars represent standard deviations. The current injection site was at X = 0. (**C**) Distribution of the energy cost per unit membrane area per AP along the axonal branching tree shown in (**A**). (**D**) Log-log plot of energy consuming power against volume in the axonal branching system when APs propagated at 4 Hz at 37 °C. Note that the scaling exponent is 0.75. Axonal volume was changed by adjusting BL from one to four. In all four systems, the diameter of the branch at the highest BL was fixed at 0.2 μm; GR = 1 for the diameter of the branches of adjacent BLs. k = 4.18 × 10^5^, b = −1.40 × 10^−5^, Adj. R-Sqr = 0.9997. (**E**) Log-log plot of energy consuming power against membrane surface in the axonal branching system. Same systems as in (D). k = 1.01, b = 2.01, Adj. R-square = 0.9996. See also Figure S1.

We next applied the energy-function method to a more complexly branched axon whose structure was that of a binary tree with GR = 1 (Fig. 3A). We found that the energy cost per unit length per AP decreased monotonically with distance from the injection site (Fig. 3A) and BL (Fig. 3B). Nonetheless, the energy cost per unit membrane area decreased sharply in the initial 400 μm before rising and then leveling off (Fig. 3C), resembling the distribution along a single-cable axon (Fig. 1D) and indicating that this distribution pattern of energy cost per unit membrane area against distance might apply to all types of axonal morphology.

Then, to examine how the energy consumption of an entire axonal branching system varies with geometry (i.e., axonal volume, membrane surface and branching complexity), we changed the geometry by increasing the total BL of the system (BL_sys_) from one to four while keeping the diameter of the highest level of child branch fixed at 0.2 μm (geometry versus BL_sys_, see Figure S1A–S1C). Interestingly, the log-log plot (Fig. 3D) shows that the dependence of the system’s metabolic rate on its volume followed an allometric scaling law with a scaling exponent 0.75 for GR = 1 here (the exponent changes from 0.3 to 2 when GR changes from 0.5 to 2). Assuming that the density of the axon (1.05 g/mL) is uniform, we found that the allometric scaling of the metabolic rate linked to AP versus the mass of the axonal tree (Figure S1E) is the same as the empirically observed scaling of the metabolic rate versus the mass of the entire organism^23^,^24^. The excellent match between our modeling and the experimental data validates the theory that uses a fractal structure model to explain the quarter-law scaling that is common in biological systems^22^. Additionally, this allometric scaling arose only when we changed the axonal tree’s volume by varying the total BL of the tree and keeping the diameter of the highest level of child branch unchanged (the results generated when the volume was changed in other ways are described in the DISCUSSION), suggesting that there may be a lower limit for the diameter of real axonal branches in any type of neuron, consistent with previous anatomical findings and computational predictions^41^; a comprehensive survey of anatomical data showed that the lower limit for AP-conducting axons is 0.08–0.2 μm in diameter, and stochastic simulations demonstrated that due to channel noise, the limiting diameter for mammalian pyramidal cell axons is 0.15 μm.

Moreover, the scaling exponent for the relationship between energy consumption and membrane surface area is close to 1 rather than 3/4 (Fig. 3E). This finding is in agreement with results from computational models of single-compartment spiking neurons^7^. This relationship can be attributed to the uniform spread of ion conductances over the membrane surface of the system. Additionally, our results showed that during AP propagation, the metabolic cost of the entire axonal branching system increased exponentially with BL_sys_ (Figure S1D).

### Effects of Ion Channel Density and Distribution on AP-related Energy Consumption

**Figure 4:**
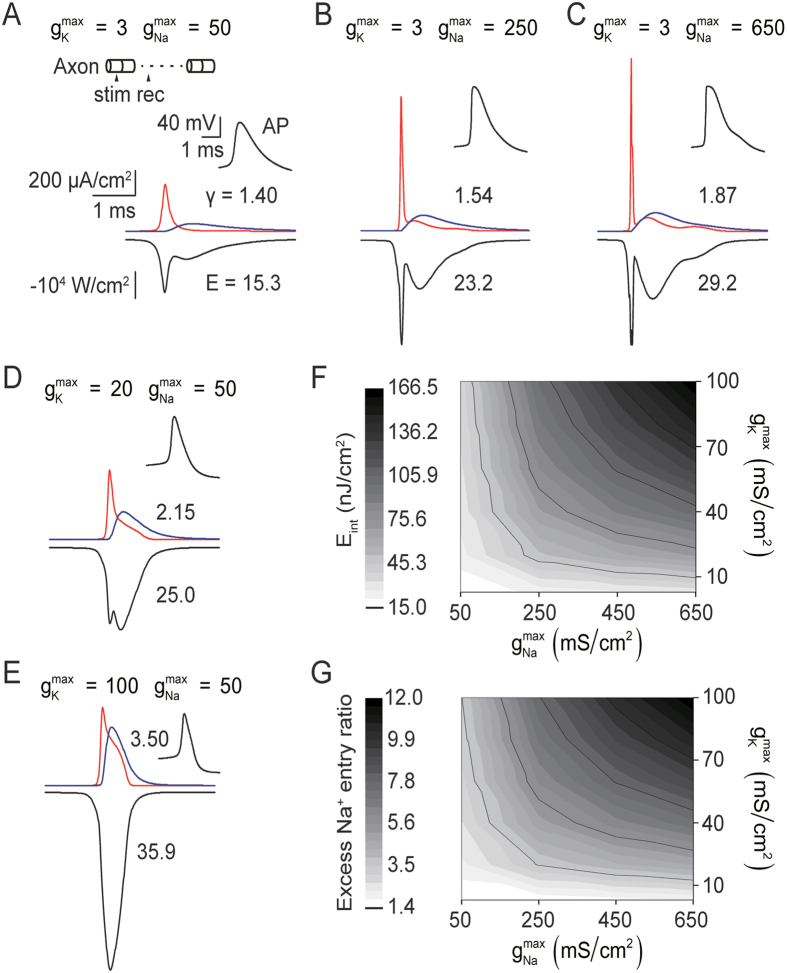
Effects of Ion Channel Density on AP-related Energy Consumption. (**A**–**E**) Traces of i_K_ (blue), -i_Na_ (red, inverted Na^+^ current) and energy cost per unit membrane area per AP (black) for APs (60 Hz, 37 °C) propagating along a single-cable axon with various combinations of maximum ion channel conductances. Numbers indicate the excess Na^+^ entry ratio (**γ**) or energy cost per unit membrane area per AP (**E**). Inset in (**A**), schematic diagram of the stimulating and recording sites along the axon (750 μm in length). (**A–C**), 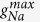 from 50 to 650 mS/cm^2^, 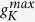 kept the same. (**A**), (**D**), (**E**), 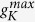 from 3 to 100 mS/cm^2^, 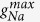 kept the same. (**F,G**) Color map of the energy cost per unit membrane area per AP at the initiation site (**F**) and the excess Na+ entry ratio at the initiation site (**G**) as a function of 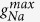 and 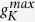. White: low cost or low ratio. Note that the minimum energy cost (**F**) and the maximum energy efficiency (**G**) were achieved when both the 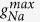 and 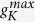 were smallest.

This section investigates how AP-related energy consumption is influenced by the density and distribution of ion channels. First, to study the impact of ion channel density, we changed the Na^+^ and K^+^ channel density of the single-cable axon (Fig. 1A) by manipulating the maximal Na^+^ and K^+^ channel conductance (g_Na_^max^ from 50 to 650 mS/cm^2^, g_k_^max^ from 3 to 100 mS/cm^2^, uniformly distributed over the axon) and then stimulated APs (60 Hz, 37 °C) that propagate along the axon. As illustrated in Fig. 4A–C, at the initiation site of the axon, when g_k_^max^ was fixed, larger g_Na_^max^ led to increased Na^+^ entry during the rising phase of the AP and increased K^+^ exit during the falling phase of the AP, increasing energy costs. A similar situation was observed when g_Na_^max^ was fixed and g_k_^max^ was changed (Fig. 4A,D,E). Because the combination of smaller g_Na_^max^ and smaller g_k_^max^ lowered energy costs and increased energy efficiency simultaneously (Fig. 4F,G), we propose that within the range of ion-channel densities sufficient for AP generation and propagation, there is an optimal combination of ion channel densities that will result in both minimal energy consumption and maximal efficiency. Actually, the effect of ion channel density found in the cable model is consistent with previous results obtained through the Na^+^-counting method for point neuronal models, including biophysical modeling and dynamic clamping of neocortical fast-spiking interneurons^8^, as well as single-compartment models of the squid giant axon and rat interneurons^7^,^42^.

**Figure 5:**
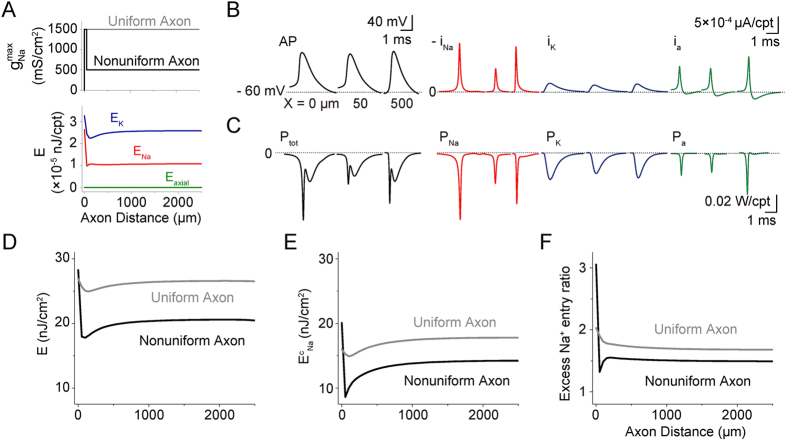
Effect of Ion Channel Distribution on AP-related Energy Consumption. (**A**) Top, uniform (grey) and nonuniform (black) Na^+^ channel distribution along the cortical axon. Bottom, variation of the main components of the total energy cost per unit membrane area per AP, i.e., the energy costs of the K^+^ (blue), Na^+^ (red) and axial (green) conductances, along the nonuniform axon when APs propagated at 60 Hz at 37 °C. (**B**) Traces of AP (black), -i_Na_ (red, inverted Na^+^ current), i_K_ (blue) and i_a_ (green) at X = 0, 50 and 500 μm. (**C**) Traces of the total energy consuming power (black) and its main components, i.e., the energy consuming power of the sodium (red), potassium (blue), and axial (green) conductances at X = 0, 50 and 500 μm. (**D,E**) Comparison of the energy cost per unit membrane area per AP along axons with nonuniform (black) and uniform (grey) Na^+^ channel expression patterns. The energy cost was obtained by the energy-function (D) and Na^+^-counting (**E**) methods. (**F**) Comparison of the excess Na^+^ entry ratio along axons with nonuniform (black) and uniform (grey) Na^+^ channel expression patterns. Note that in (**D–F**), the values of the nonuniform axon were lower than those of the uniform axon, except in the initial 50 μm.

Next, to investigate the influence of ion channel distribution, we carried out simulations in the unbranched axon with a nonuniform Na^+^ channel expression pattern (Fig. 5A, top) and then compared the results with those of the uniform axon. In the nonuniform axon, the initial 50 μm, where g_Na_^max^ is three times larger than the value for the rest of the axon, showed a dramatic decrease in Na^+^ entry (Fig. 5B, red, note the amplitude) and K^+^ exit during an AP (blue). This finding accounts for the sharp decrease in two of the main components of energy expenditure, E_Na_ (62% drop) and E_K_ (33% drop) (see Fig. 5A, bottom; Fig. 5C), and consequently, the significant decrease in total energy expenditure (36% drop, see Fig. 5C,D, black). Noticeably, both the energy cost (Fig. 5D, black) and the excess Na^+^ entry ratio (Fig. 5F, black) reached minimum values right at the end of the high Na^+^ density area. Importantly, comparing the energy costs along the nonuniform axon and the uniform one, based on either the energy-function (Fig. 5D) or Na^+^-counting (Fig. 5E) method reveals that the nonuniform Na^+^ channel pattern resulted in an energy savings of approximately 20% for the total axon. Similarly, comparing the excess Na^+^ entry ratios (Fig. 5F) shows that the nonuniform Na^+^ channel pattern enhanced energy efficiency by approximately 10%, with a saturated Na^+^ entry-ratio value of approximately 1.5 compared with the uniform pattern (in gray line). The low energy usage and high energy efficiency of the nonuniform pattern concluded here by the simulations could be a novel prediction for further experimental investigation. It actually suggests a nontrivial rule that actual neuronal axons may have nonuniform distributed ionic channel distributions in an energy efficient strategy.

## Discussion

Based on the energy function used to estimate the metabolic cost of APs in HH point neuron models^19^,^20^,^21^, we established the energy-function method for the cable model of axons. Through this approach, we provided the first demonstration that AP propagation requires 15% more energy at the initiation site (Fig. 1D, inset) than the amount predicted by the point model. In addition, the allometric scaling relationship between the total energetic rate of the axonal branching tree (*P*_*tot*_) versus the axonal volume (*V*), 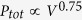, suggested that the metabolic rate of a single neuron is very likely to scale to the 3/4 power of its mass, just as the metabolic rate of an entire organism scales with its body mass in animals^23^, plants and microbes^24^. Additionally, the allometric scaling relationship suggests an invariant minimum diameter for axonal branches in any type of neuron; this suggestion is supported by anatomical findings and stochastic simulations that take channel noise into account^41^. Moreover, by enabling the energy costs to be precisely distributed over the complicated branching pattern (Fig. 3A), this method can be applied to branched dendrites as well.

### Advantages of the Cable Energy Function

The cable energy function derived here provides an accurate method for calculating the energy cost of electric signals conducted in any type of neuron with an axonal or dendritic arbor. This theoretical framework allows us to estimate the energy used by cortical axons with many types of ion channels more accurately than is possible with the Na^+^-counting method, especially in the presence of some subtypes of Ca^2+^ and K^+^ channels. By taking into account all of the energy-consuming components in the cable equation, including the axial and transmembrane leak currents, the energy function provides a precise evaluation at any axonal distance and any time (see METHODS). The distribution of energy usage among all of the energy-consuming components in the cortical neuron shows that potassium and sodium conductances consumed the most energy; together, these conductances accounted for 84.4% of the total energy usage in the cable model (Figure S2) and 96% in the point model. This finding is comparable to the result of the point model of the squid giant neuron^19^.

In contrast, the Na^+^-counting method tends to underestimate energy consumption (Fig. 1F). In fact, because it is based on the number of ATP molecules required by Na^+^/K^+^-ATPase pumps to restore the Na^+^ and K^+^ concentration gradients after an AP, this method merely calculates the energy cost related to the transmembrane flowing of Na^+^ and K^+^ ions. However, even when the energy usage contributed by sodium and potassium channels only was considered for the energy-function method, the energy usage estimate derived from the Na^+^-counting method was 20% lower for the cortical neuron cable model and 32% lower for the point model. More importantly, we have also examined how the discrepancy between the two methods is influenced by the parameters, i.e. spiking rate, length of cable, temperature and g^max^. The discrepancy is calculated as 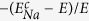, where 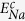 and *E* are the results of the sodium counting method and our approach, respectively. When one factor was examined, other factors were kept the same. Our results show that these factors have limited effects on the discrepancy (<10%), indicating that the discrepancy is quite stable. The detailed results are as follows. First, when the spiking rate increased from 5 Hz to 125 Hz, the discrepancy of the point model and the cable model increased 7% (39~46%) and 1% (37~38%), respectively. Second, when the length of the cable was ≤3000 um, the discrepancy was within the range of 27~38%. Third, when the temperature was in the range of 6 °C~42 °C, the discrepancy was within the range of 32~37%. Fourth, larger 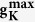 and 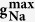 leads to larger discrepancy, and within the range of 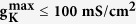 and 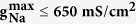, the discrepancy is within the range of 30~40%.

For a deeper understanding of how the two methods calculate energy differently, see Methods. In addition, the energy-function method predicts some interesting phenomena. One example is the nonuniform Na^+^ channel expression pattern, which has been observed experimentally and was demonstrated to be vital for axonal signaling functions in previous modeling studies^9^,^43^. The natural beauty of the computational modeling is that it could provide novel prediction to instruct new experiment investigation and confirmation. The model conclusion of high energy efficiency with nonuniform ionic channel distribution could be a nontrivial point for new experimental examination. Other predictions, such as the prediction that longer axonal length leads to higher energy consumption (Fig. 1D) and the prediction that there is an optimal combination of ion channel densities that will minimize metabolic cost (Fig. 4F,G), need to be experimentally validated. Furthermore, this analytical approach can be used to make predictions regarding a diverse range of neurons because it can be applied to any HH-type neuron model, such as one where Ca^+^ channels play an important role in AP generation, simply by deriving the energy function for that model.

### Allometric Scaling of Energy Versus Volume

Biological systems have evolved branching networks for information flow and energy transport. Experimental^23^,^44^,^45^,^46^ and theoretical studies^22^,^47^,^48^ have shown that the metabolic rate of a given biological system generally scales with the 3/4 power of system volume or mass, and the potential mechanism predicted a space-filling, fractal-like branching pattern with a minimized energy distribution^22^. In this paper, we also examined the relationship between the metabolic rate and volume of cortical axons. To do this, the diameter of the final branch level was set to an invariant value of 0.2 μm, as reported by experiments. This setting ensured a realistic value for the diameters of axonal branches in pyramidal cells^41^; these values varied from 0.2 to 1.27 μm (Fig. 3A, inset, note that GR = 1 for the diameter of branches at adjacent BLs). The model analysis predicted that allometric scaling with a scaling exponent of 0.75 arose only when the tree’s volume was changed by varying the branch level while keeping the diameter of the final branch level constant. When we kept the diameter of the lowest level of parent branch constant in trees of different branch level (Figure S1F) or when we changed the volume of the single uniform axon by changing the length of each branch (Figure S1G), the scaling relation could not be obtained. Interestingly, our setting of the model is consistent with the main assumption of the general theory used to explain the widely used quarter-power scaling, i.e., keeping the final branch of the fractal network a size-invariant unit^22^.

The power law relation between the energy consumption of the whole axonal architecture and the level of branching (Figure S1D) was also observed in computational models of the bifurcating axonal arbor of dopamine neurons of the substantia nigra pars compacta^49^. The high energy demand of the massive axonal arbors of these neurons, which was confirmed by the computational work, was proposed as a factor in the selective vulnerability of these dopamine neurons to Parkinson’s disease^49^.

### Implications of the Analytical Approach

With extended applications from axons to more complex neuronal systems, this analytical approach could play a significant role in the estimation of energy expenditure, from subcellular to whole-organism levels. For instance, by applying this approach to the morphology-based HH neuron model that was built on experimental data, we can obtain a more realistic subcellular distribution of energy use, compared to the one that was first obtained through calculations of Na^+^ influx^1^. Similarly, the energy function could be used to replace previous estimates of the elevation of AP-related energy, which is highly dependent on the excess Na^+^ entry ratio, within the neural signaling energy budgets that have been constructed for grey matter^2^,^4^ and white matter^3^.

Furthermore, this analytical method of energy calculation can be used to investigate factors that influence energy consumption and reveal trade-offs between energetic constraints and signaling performance. In this study, we examined the impact of axonal geometry, AP propagation state, and ion channel density. Other factors that strongly affect AP conduction speed and spiking frequency^7^,^8^,^9^,^10^,^11^,^16^, such as information rate^6^,^7^,^50^ and ion channel kinetics, are worth re-investigating using the analytical approach to confirm previous conclusions.

Moreover, this analytical approach provides a valuable way of understanding energetic constraints on neuronal morphology. For instance, the repression of axonal branching, which is a crucial regulatory mechanism that can counteract positive cues to evoke branching^51^, might also be an energy-saving strategy because metabolic costs linearly increase with increasing surface area of the axon branching system (Fig. 3E). In addition, how the growth of an axonal process is coordinated with the retraction of another is unknown on a molecular level^51^, and from an energy consumption standpoint, the repression of axonal branching might help to achieve physiological function within the limits of available energy resources. Furthermore, branch points, which are thought to represent a powerful process that filters communication with postsynaptic neurons^32^, might also have an energy-conserving function. Our results, which demonstrate a reduction in metabolic cost when conduction fails compared to when it succeeds (Fig. 2D,E), support the idea first proposed by Hallermann *et al*. that there is a tradeoff between energy minimization and the reliability of AP propagation^1^. In their study, successful propagation of APs into collaterals of the main axon depended on the inactivation kinetics of Na^+^ channels, while in ours, it depended on the geometrical relationship between the diameters of the branches before and after the branch point. Additionally, a previous study^52^ showed that the location of somata relative to the dendritic and axonal trees affects signal attenuation and hence metabolic costs. This result can be reinvestigated through direct energy calculations based on the energy function.

We believe this analytical approach will provide new insight into the metabolic costs of brain signaling, which is considered to be energetically expensive compared with other metabolic processes, although it is remarkably efficient compared with computers. On the one hand, the brain accounts for 20% of the body’s total resting oxygen consumption^53^,^54^,^55^ despite accounting for only 2% of body mass. This limited energy supply imposes serious metabolic constraints on brain function and evolution, including constraints on the density and size of neurons^7^,^56^,^57^ and on functional connectivity^58^, network activity^59^, and coding strategies^60^,^61^,^62^,^63^. On the other hand, the human brain’s power consumption is approximately 20 W^64^, which is merely 0.00001% of the power of today’s most advanced supercomputers^65^. Finally, accurate estimation of the metabolic cost will improve the interpretation of functional magnetic resonance imaging data^12^,^13^,^14^.

## Methods

### Cable Model of Hodgkin-Huxley-type Cortical Axon

To describe AP propagation along an axon, the cable equation that describes the flow of ion currents along the axon needs to be derived^66^,^67^. Figure 1A gives the equivalent circuit of the cable model. During AP propagation, the membrane potential, *V*(*x*, *t*), changes along the *x*-axis,


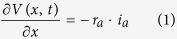


where 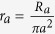, *R*_a_ = 0.15 kΩ · cm is the axial intracellular resistivity, *a* is the axon radius, and *i*_*a*_ (μA) is the axial current. According to Kirchhoff’s current law, *i*_*a*_ varies along the x-axis,


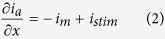


where *i*_*m*_ is the membrane-crossing current and *i*_*stim*_ is the AP-stimulating current. The membrane-crossing current *i*_*m*_ consists of the capacitance and the ion currents,


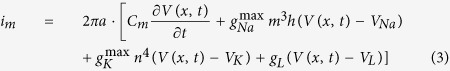


where *C*_*m*_ = 0.75 μF/cm^2^ is the membrane capacitance; 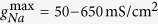, 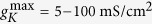, and *g*_*L*_ = 0.033 mS/cm^2^ are the maximal sodium, maximal potassium, and leak conductance per unit membrane area, respectively; and *V*_*Na*_ = 60 mV, *V*_*K*_ = −90 mV, and *V*_*L*_ = −70 mV are the reversal potentials of the sodium, potassium, and leak channels, respectively. The gate variables *m*, *h*, and *n* are dimensionless activation and inactivation variables, which describe the activation and inactivation processes of the sodium and potassium channels and are governed by the following differential equations^18^,


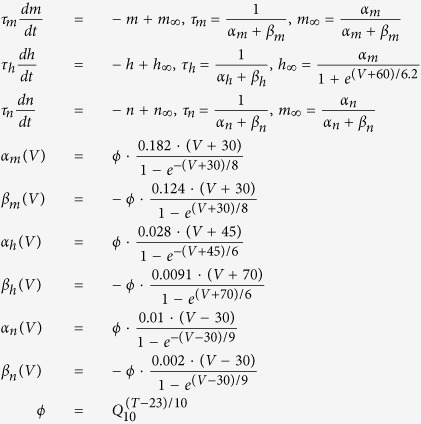


where *ϕ* regulates the temperature dependence, with Q_10_ = 2.3.

Combining Equations 1, 2, 3, we obtain,


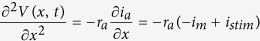


i.e., the cable equation,


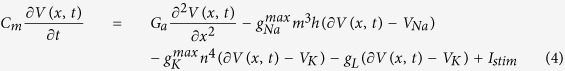


where 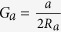 is the axial conductance and *I*_*stim*_ = *i*_*stim*_/2πa (μA/cm^2^).

### Energy Consumption of AP Propagation in the Cable Model

Inspired from the procedure applied to the classical HH model to compute the electrochemical energy involved in the dynamics of point model of single neuronal circuit^19^ we plan to derive energy function of neuronal axon in the case of multi-compartment neuronal model as the method used in previous reference^19^. In the entire equivalent circuit of the cortical cable model, the batteries provide electrical energy for the capacitors and the conductors. According to the energy conservation law, the summation of energy provided by all batteries in the cable (*H*_*b*_^*all*^, kJ), the stored energy in capacitance (*H*_*C*_^*all*^, kJ), the input external stimulus energy (*H*_*s*_^*all*^, kJ), and the dissipated energy by all conductors in the cable (*H*_*g*_^*all*^, kJ) should be equal to 0, i.e.,


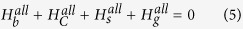


or


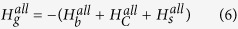


where the battery energy *H*_*b*_^*all*^ = *H*_*K*_^*all*^ + *H*_*Na*_^*all*^ + *H*_*L*_^*all*^ with batteries energy from potassium channel, sodium channel, and leaky channel, and are represented as following, respectively,


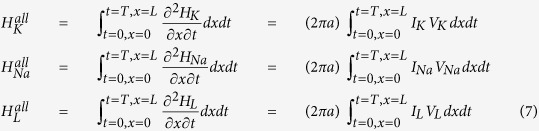


The role of the capacitance is to store energy from the battery supplier, not to consume energy, the total energy of the capacitors of the axon cable is


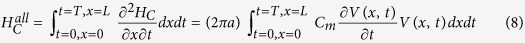


The total stimulus energy *H*_*s*_^*all*^ induced by stimulus *I*_*s*_ is expressed as


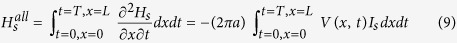


The total consumed energy *H*_*g*_^*all*^ by all dissipated items could be calculated by summation of battery energy, capacitance energy and the stimulus energy based on Eq. 6. To substitute Eqs. 7, 8, 9 into Eq. 6, we have


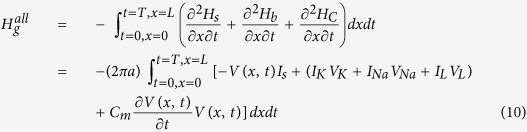


To substitute Eq. 4 into Eq. 10, the following equation is derived


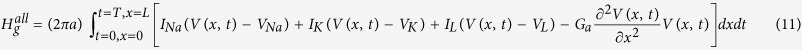


To substitute each item, the energy consumption equation of cable model (let *E* = *H*_*g*_) is derived as,


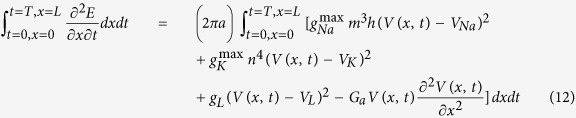


In summary, our approach is based on calculation of energy dissipation through conductances of the cable (including the axial conductance). In methods, we demonstrate the mathematical consistency of this with energy conservation and calculation of energy supplied by the ionic reversal potentials (and stimulus). During AP firing, the summation of external stimulus energy, the ion battery energy and the capacitor storing energy should be equal to the dissipated energy by conductors. Within the circuit, ion batteries supply energy, capacitors store energy while only conductors consume energy for action potential generation and initiation. Actually, computing the ATP consumption of the Na+/K+-ATPase pumps is a crude way to estimate the energy discharged from the batteries during AP firing. The explanation is as follows. The amount of energy stored in the ion batteries depends on the ion concentration gradients across the membrane. During AP firing, the membrane-crossing of the ions decreases the concentration gradients, which decreases the energy stored in the ion batteries. Conversely, after AP firing, the Na^+^-K^+^-ATPase recharges the ion batteries by restoring the ion concentration gradients to the equilibrium level. The ATP used in this restoring process is supposed to be the energy required to recharge the ion batteries after AP firing, which is numerically equal to the energy discharged from the batteries during AP firing.

Based on the explanation above, there are two main reasons for the inaccuracy of the sodium counting approach. First, it is not an accurate way to estimate the energy dissipated by the conductors. Second, even as a way to compute the energy discharged by the ion batteries, it only gives a coarse estimation by calculating the ATP molecules by the ratio of the number of pumped out sodium ions to needed ATP molecules, i.e. 3:1. According to our simulation, this coarse estimation is significantly lower than the direct calculation of the energy discharged from the batteries. Furthermore, whether the ATP calculating approach should be based on Na^+^-counting or K^+^-counting is controversial. Some believed that calculating the ATP molecules by the ratio of the number of pumped in potassium ions to needed ATP molecules, i.e. 2:1, is more accurate^5^. Actually, those two methods give different results.

From the biological perspective, we want to emphasize that, besides the sodium currents which have been widely recognized as making important contribution to the metabolic cost of AP firing, the potassium currents also contribute significantly to the cost. The potassium currents not only cause energy dissipation when flowing out of the membrane to restore the membrane potential, but also may be a part of the axial currents that result in the energy dissipation of propagating AP along the axon.

## Additional Information

**How to cite this article**: Ju, H. *et al*. Cable energy function of cortical axons. *Sci. Rep.* **6**, 29686; doi: 10.1038/srep29686 (2016).

## Supplementary Material

Supplementary Information

## References

[b1] Hallermann S., de Kock C. P., Stuart G. J. & Kole M. H. State and location dependence of action potential metabolic cost in cortical pyramidal neurons. Nat Neurosci 15, 1007–1014, 10.1038/nn.3132 (2012). 22660478

[b2] Attwell D. & Laughlin S. B. An energy budget for signaling in the grey matter of the brain. J Cereb Blood Flow Metab 21, 1133–1145, 10.1097/00004647-200110000-00001 (2001). 11598490

[b3] Harris J. J. & Attwell D. The energetics of CNS white matter. J Neurosci 32, 356–371, 10.1523/JNEUROSCI.3430-11. (2012). 22219296 PMC3272449

[b4] Howarth C., Gleeson P. & Attwell D. Updated energy budgets for neural computation in the neocortex and cerebellum. J Cereb Blood Flow Metab 32, 1222–1232, 10.1038/jcbfm.2012.35 (2012). 22434069 PMC3390818

[b5] Crotty P., Sangrey T. & Levy W. B. Metabolic energy cost of action potential velocity. J Neurophysiol 96, 1237–1246, 10.1152/jn.01204.2005 (2006). 16554507

[b6] Niven J. E., Anderson J. C. & Laughlin S. B. Fly photoreceptors demonstrate energy-information trade-offs in neural coding. PLoS biology 5, e116, 10.1371/journal.pbio.0050116 (2007). 17373859 PMC1828148

[b7] Sengupta B., Faisal A. A., Laughlin S. B. & Niven J. E. The effect of cell size and channel density on neuronal information encoding and energy efficiency. J Cereb Blood Flow Metab 33, 1465–1473, 10.1038/jcbfm.2013.103 (2013). 23778164 PMC3764378

[b8] Hasenstaub A., Otte S., Callaway E. & Sejnowski T. J. Metabolic cost as a unifying principle governing neuronal biophysics. Proc Natl Acad Sci USA 107, 12329–12334, 10.1073/pnas.0914886107 (2010). 20616090 PMC2901447

[b9] Schmidt-Hieber C. & Bischofberger J. Fast sodium channel gating supports localized and efficient axonal action potential initiation. J Neurosci 30, 10233–10242, 10.1523/JNEUROSCI.6335-09.2010 (2010). 20668206 PMC6633381

[b10] Lewis J. E., Gilmour K. M., Moorhead M. J., Perry S. F. & Markham M. R. Action potential energetics at the organismal level reveal a trade-off in efficiency at high firing rates. J Neurosci 34, 197–201, 10.1523/JNEUROSCI.3180-13.201434/1/197 (2014). 24381281 PMC3866484

[b11] Hu H. & Jonas P. A supercritical density of Na(+) channels ensures fast signaling in GABAergic interneuron axons. Nat Neurosci 17, 686–693, 10.1038/nn.3678 (2014). 24657965 PMC4286295

[b12] Logothetis N. K. What we can do and what we cannot do with fMRI. Nature 453, 869–878, 10.1038/nature06976 (2008). 18548064

[b13] Mangia S. . Metabolic and hemodynamic events after changes in neuronal activity: current hypotheses, theoretical predictions and *in vivo* NMR experimental findings. J Cereb Blood Flow Metab 29, 441–463, 10.1038/jcbfm.2008.134 (2009). 19002199 PMC2743443

[b14] Magistretti P. J. & Allaman I. A Cellular Perspective on Brain Energy Metabolism and Functional Imaging. Neuron 86, 883–901, 10.1016/j.neuron.2015.03.035 (2015). 25996133

[b15] Hodgkin A. The optimum density of sodium channels in an unmyelinated nerve. Philos Trans R Soc Lond B Biol Sci 270, 297–300 (1975). 238229 10.1098/rstb.1975.0010

[b16] Carter B. C. & Bean B. P. Sodium entry during action potentials of mammalian neurons: incomplete inactivation and reduced metabolic efficiency in fast-spiking neurons. Neuron 64, 898–909, 10.1016/j.neuron.2009.12.011 (2009). 20064395 PMC2810867

[b17] Alle H., Roth A. & Geiger J. R. Energy-efficient action potentials in hippocampal mossy fibers. Science 325, 1405–1408, 10.1126/science.1174331 (2009). 19745156

[b18] Yu Y., Hill A. P. & McCormick D. A. Warm body temperature facilitates energy efficient cortical action potentials. PLoS Comput Biol 8, e1002456, 10.1371/journal.pcbi.1002456PCOMPBIOL-D-11-01127 [pii] (2012). 22511855 PMC3325181

[b19] Moujahid A., d’Anjou A., Torrealdea F. J. & Torrealdea F. Energy and information in Hodgkin-Huxley neurons. Phys Rev E 83, 10.1103/PhysRevE.83.031912 (2011). 21517530

[b20] Moujahid A. & d’Anjou A. Metabolic efficiency with fast spiking in the squid axon. Front Comput Neurosci 6, 95, 10.3389/fncom.2012.00095 (2012). 23162461 PMC3498622

[b21] Moujahid A., D’Anjou A. & Grana M. Energy demands of diverse spiking cells from the neocortex, hippocampus, and thalamus. Front Comput Neurosci 8, 41, 10.3389/fncom.2014.00041 (2014). 24782749 PMC3986563

[b22] West G. B., Brown J. H. & Enquist B. J. A general model for the origin of allometric scaling laws in biology. Science 276, 122–126 (1997). 9082983 10.1126/science.276.5309.122

[b23] Martin R. D. Relative brain size and basal metabolic rate in terrestrial vertebrates. Nature 293, 57–60 (1981). 7266659 10.1038/293057a0

[b24] Hemmingsen A. M. The relation of standard. (basal) energy metabolism to total fresh weight of living organisms. Rep. Steno Mem. Hosp. (Copenhagen) 4, 1–58 (1950).

[b25] Rall W. Cable theory for dendritic neurons. In Methods in neuronal modeling. 9–62 (MIT press 1989).

[b26] Yu Y., Shu Y. & McCormick D. A. Cortical Action Potential Backpropagation Explains Spike Threshold Variability and Rapid-Onset Kinetics. J Neurosci 28, 7260–7272, 10.1523/jneurosci.1613-08.2008 (2008). 18632930 PMC2664555

[b27] Shu Y., Yu Y., Yang J. & McCormick D. A. Selective control of cortical axonal spikes by a slowly inactivating K+ current. Proc Natl Acad Sci USA 104, 11453–11458, 10.1073/pnas.0702041104 (2007). 17581873 PMC2040919

[b28] Shu Y., Duque A., Yu Y., Haider B. & McCormick D. A. Properties of action-potential initiation in neocortical pyramidal cells: evidence from whole cell axon recordings. J Neurophysiol 97, 746–760, 00922.2006 (2007). 17093120 10.1152/jn.00922.2006

[b29] McCormick D. A., Shu Y. & Yu Y. Neurophysiology: Hodgkin and Huxley model–still standing? Nature 445, E1–E2, discussion E2–E3, nature05523 (2007). 17203021 10.1038/nature05523

[b30] Shu Y., Hasenstaub A., Duque A., Yu Y. & McCormick D. A. Modulation of intracortical synaptic potentials by presynaptic somatic membrane potential. Nature 441, 761–765, nature04720 (2006). 16625207 10.1038/nature04720

[b31] Yu Y., Shu Y. & McCormick D. A. Cortical action potential backpropagation explains spike threshold variability and rapid-onset kinetics. J Neurosci 28, 7260–7272, 10.1523/JNEUROSCI.1613-08.2008 (2008). 18632930 PMC2664555

[b32] Debanne D., Campanac E., Bialowas A., Carlier E. & Alcaraz G. Axon Physiology. Physiol Rev 91, 555–602, 10.1152/physrev.00048.2009 (2011). 21527732

[b33] Parnas I. & Segev I. A mathematical model for conduction of action potentials along bifurcating axons. J Physiol 295, 323–343 (1979). 521942 10.1113/jphysiol.1979.sp012971 PMC1279048

[b34] Goldstein S. S. & Rall W. Changes of action potential shape and velocity for changing core conductor geometry. Biophys J 14, 731–757, 10.1016/S0006-3495(74)85947-3 (1974). 4420585 PMC1334570

[b35] Maia P. D. & Kutz J. N. Identifying critical regions for spike propagation in axon segments. J Comput Neurosci 36, 141–155, 10.1007/s10827-013-0459-3 (2014). 23818067

[b36] Grossman Y., Parnas I. & Spira M. E. Differential conduction block in branches of a bifurcating axon. J Physiol 295, 283–305 (1979). 521937 10.1113/jphysiol.1979.sp012969 PMC1279046

[b37] Grossman Y., Parnas I. & Spira M. E. Mechanisms involved in differential conduction of potentials at high frequency in a branching axon. J Physiol 295, 307–322 (1979). 521940 10.1113/jphysiol.1979.sp012970 PMC1279047

[b38] Westerfield M., Joyner R. W. & Moore J. W. Temperature-sensitive conduction failure at axon branch points. J Neurophysiol 41, 1–8 (1978). 621537 10.1152/jn.1978.41.1.1

[b39] Rall W. Branching dendritic trees and motoneuron membrane resistivity. Exp Neurol 1, 491–527 (1959). 14435979 10.1016/0014-4886(59)90046-9

[b40] Rall W. Theory of physiological properties of dendrites. Ann N Y Acad Sci 96, 1071–1092 (1962). 14490041 10.1111/j.1749-6632.1962.tb54120.x

[b41] Faisal A. A., White J. A. & Laughlin S. B. Ion-channel noise places limits on the miniaturization of the brain’s wiring. Curr Biol 15, 1143–1149, 10.1016/j.cub.2005.05.056 (2005). 15964281

[b42] Sengupta B., Stemmler M., Laughlin S. B. & Niven J. E. Action potential energy efficiency varies among neuron types in vertebrates and invertebrates. PLoS Comput Biol 6, e1000840, 10.1371/journal.pcbi.1000840 (2010). 20617202 PMC2895638

[b43] Kole M. H. . Action potential generation requires a high sodium channel density in the axon initial segment. Nat Neurosci 11, 178–186, 10.1038/nn2040 (2008). 18204443

[b44] Glazier D. S. Beyond the ‘3/4-power law’: variation in the intra- and interspecific scaling of metabolic rate in animals. Biol Rev Camb Philos Soc 80, 611–662, 10.1017/S1464793105006834 (2005). 16221332

[b45] Kleiber M. Body Size and Metabolic Rate. Physiol Rev 27, 511–541 (1947). 20267758 10.1152/physrev.1947.27.4.511

[b46] White C. R. & Seymour R. S. Allometric scaling of mammalian metabolism. J Exp Biol 208, 1611–1619, 10.1242/jeb.01501 (2005). 15855392

[b47] Brown J. H., Gillooly J. F., Allen A. P., Savage V. M. & West G. B. Toward A Metabolic Theory of Ecology. Ecology 85, 1771–1789, 10.1890/03-9000 (2004).

[b48] Gillooly J. F., Brown J. H., West G. B., Savage V. M. & Charnov E. L. Effects of size and temperature on metabolic rate. Science 293, 2248–2251, 10.1126/science.1061967 (2001). 11567137

[b49] Pissadaki E. K. & Bolam J. P. The energy cost of action potential propagation in dopamine neurons: clues to susceptibility in Parkinson’s disease. Front Comput Neurosci 7, 13, 10.3389/fncom.2013.00013 (2013). 23515615 PMC3600574

[b50] Levy W. B. & Baxter R. A. Energy efficient neural codes. Neural Comput 8, 531–543 (1996). 8868566 10.1162/neco.1996.8.3.531

[b51] Acebes A. & Ferrus A. Cellular and molecular features of axon collaterals and dendrites. Trends Neurosci 23, 557–565 (2000). 11074265 10.1016/s0166-2236(00)01646-5

[b52] Hesse J. & Schreiber S. Externalization of neuronal somata as an evolutionary strategy for energy economization. Curr Biol 25, R324–R325, 10.1016/j.cub.2015.02.024 (2015). 25898099

[b53] Sokoloff L. In Handbook of Physiology, Section I, Neurophysiology, vol. 3 (eds Field John, Magoun Horace Winchell, & Hall Victor E ) 1843–1864 (American Physiological Society, 1960).

[b54] Kety S. S. In Metabolism of the Nervous System (ed Richter Derek ) 221–237 (Pergamon, 1957).

[b55] Rolfe D. F. & Brown G. C. Cellular energy utilization and molecular origin of standard metabolic rate in mammals. Physiol Rev 77, 731–758 (1997). 9234964 10.1152/physrev.1997.77.3.731

[b56] Karbowski J. Thermodynamic constraints on neural dimensions, firing rates, brain temperature and size. J Comp Neurosci 27, 415–436, 10.1007/s10827-009-0153-7 (2009). 19415477

[b57] Herculano-Houzel S. Scaling of brain metabolism with a fixed energy budget per neuron: implications for neuronal activity, plasticity and evolution. PLoS One 6, e17514, 10.1371/journal.pone.0017514 (2011). 21390261 PMC3046985

[b58] Tomasi D., Wang G. J. & Volkow N. D. Energetic cost of brain functional connectivity. Proc Natl Acad Sci USA 110, 13642–13647, 10.1073/pnas.1303346110 (2013). 23898179 PMC3746878

[b59] Kann O. The energy demand of fast neuronal network oscillations: insights from brain slice preparations. Front Pharmacol 2, 90, 10.3389/fphar.2011.00090 (2011). 22291647 PMC3254178

[b60] Laughlin S. B. Energy as a constraint on the coding and processing of sensory information. Curr Opin Neurobiol 11, 475–480 (2001). 11502395 10.1016/s0959-4388(00)00237-3

[b61] Balduzzi D., Ortega P. A. & Besserve M. Metabolic Cost as an Organizing Principle for Cooperative Learning. Advances in Complex Systems 16, 1350012, 10.1142/s0219525913500124 (2013).

[b62] Kostal L., Lansky P. & McDonnell M. D. Metabolic cost of neuronal information in an empirical stimulus-response model. Biol Cybern 107, 355–365, 10.1007/s00422-013-0554-6 (2013). 23467914

[b63] Lennie P. The Cost of Cortical Computation. Curr Biol 13, 493–497, 10.1016/s0960-9822(03)00135-0 (2003). 12646132

[b64] Aiello L. C. & Wheeler P. The expensive-tissue hypothesis: the brain and the digestive system in human and primate evolution. Curr Anthropology 36, 199–221 (1995).

[b65] Scogland T. . Node variability in large-scale power measurements: perspectives from the Green500, Top500 and EEHPCWG. In Proceedings of the International Conference for High Performance Computing, Networking, Storage and Analysis 74 (ACM, 2015).

[b66] Gabbiani F. & Cox S. J. In Mathematics for Neuroscientists (eds Gabbiani Fabrizio & Cox Steven J ) 119–141 (Academic Press, 2010).

[b67] Johnston D. & Wu S. M.-S. In Foundations of cellular neurophysiology 143–181 (MIT Press, Cambridge, MA, 1995).

