# Supplementary material for: Cable energy function of cortical axons
Source: Sci Rep. 2016 Jul 21;6:29686. doi: 10.1038/srep29686 (PMC4954988; doi:10.1038/srep29686)
Supplement: Supplementary Information [file srep29686-s1.pdf]

Cable energy function of cortical axons

Huiwen Ju[1], Michael L. Hines[2] and Yuguo Yu[1]*

[1]School of Life Science and the Collaborative Innovation Center for Brain Science,

Center for Computational Systems Biology, Fudan University, Shanghai, China

200433

[2]Department of Neuroscience, Yale University School of Medicine, New Haven, CT

06520, USA

Supplemental Material

Figure S1. Related to Figure 3**.**

(A-C) The number of branch points (A), membrane surface (B) and volume (C) increase exponentially with branching level of the axonal branching system. See inset of (D) or inset of Figure 3D for schematic diagrams of the systems. BL_{sys} represents branching level of the system.

(D and E) Log-log plot of the energy consuming power of the system against BL_{sys} (D) and against the mass of the system (E), when APs propagated at 4 Hz at 37 ℃. In all four systems (see inset of (D)), diameter of the branches at the highest branching level (BL) fixed at 0.2 µm; GR = 1 for diameter of the branches at adjacent BLs. In (E), mass = volume × density, density = 1.05g/mL; k = 6.04×10^5, b = $- 7.27 \times 10^{-5}$, Adj. R-Sqr = 0.9999.

(F and G) Log-log plot of the energy consuming power of the system against the volume which was changed in other ways. In (F), volume of the system was changed by BL_{sys} while the diameter of the branch at BL = 0 was kept the same. Inset of (F), volume of the system versus BL_{sys}. In (G), the length of a single cable was changed. Note that the scaling exponent in the formula for the energy consuming power against the volume in (F) and (G) is not the 3/4 power law.

Figure S2. Related to Figure 1.

Left, total energy consumed per AP per compartment (black) and its main components, i.e. the one consumed by K^+ (blue), Na^+ (red) and axial (green) conductance, varied with axon distance in the cable model shown in Figure 1A. "cpt" represents compartment. Right, pie chart of the proportion of energetic cost per AP in the cable model of a cortical axon, comparing contribution by K^+, Na^+, leak and axial conductance.

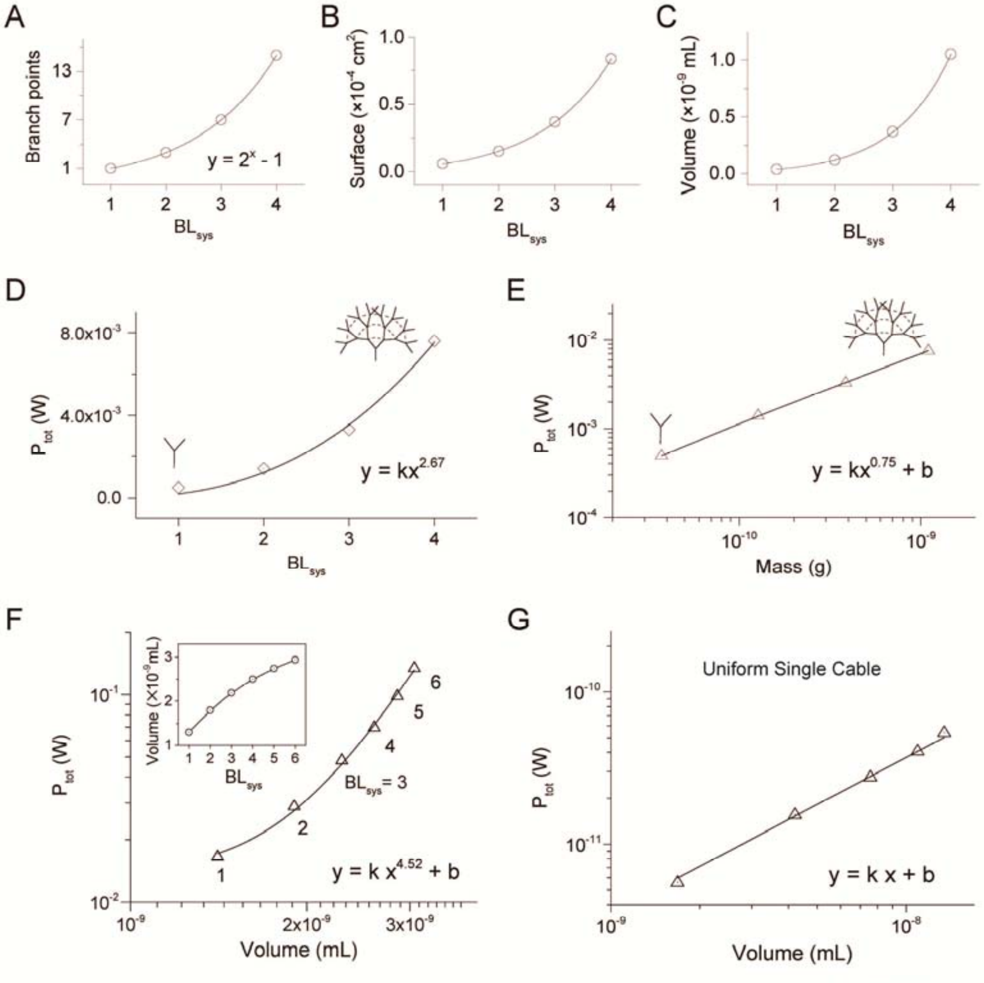

Figure S1

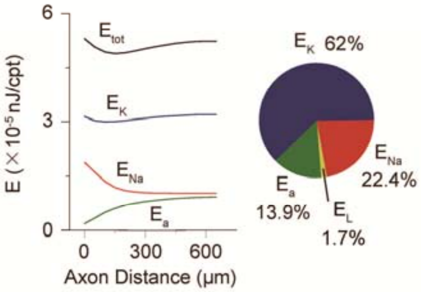

Figure S2
